# The Added Value of Advanced Echocardiography for the Morpho-Functional and Prognostic Evaluation of the Right Heart in Dilated Cardiomyopathy: Do Not Forget about the Right Atrium

**DOI:** 10.3390/jcm13051400

**Published:** 2024-02-28

**Authors:** Călin-Dinu Hădăreanu, Diana-Ruxandra Hădăreanu, Flavia-Mihaela Stoiculescu, Victor-Cornel Raicea, Georgică-Costinel Târtea, Cristina Florescu, Răzvan Ilie Radu, Ionuț Donoiu

**Affiliations:** 1 Department of Cardiology, University of Medicine and Pharmacy of Craiova, 2 Petru Rares St., 200349 Craiova, Romania; 2 Department of Cardiovascular Surgery, Clinical Emergency County Hospital of Craiova, 1 Tabaci St., 200642 Craiova, Romania; 3 Department of Cardiology, Clinical Emergency County Hospital of Craiova, 1 Tabaci St., 200642 Craiova, Romania; 4 Department of Physiology, University of Medicine and Pharmacy of Craiova, 2 Petru Rares St., 200349 Craiova, Romania; 5 Department of Cardiology, Filantropia Clinical Hospital, 28 Sararilor St., 200516 Craiova, Romania; 6 Department of Interventional Cardiology, Prof. Dr. C. C. Iliescu Emergency Institute for Cardiovascular Diseases, 258 Fundeni St., 022328 Bucharest, Romania; 7 Department of Cardiology, Carol Davila University of Medicine and Pharmacy, 8 Eroii Sanitari Bld., 050474 Bucharest, Romania

**Keywords:** dilated cardiomyopathy, right atrium, right ventricle, right heart, speckle-tracking echocardiography, three-dimensional echocardiography, heart failure

## Abstract

(1) Introduction and Aims: Right ventricular (RV) remodeling significantly impacts the prognosis of dilated cardiomyopathy (DCM) patients, and right atrial (RA) size and function are still often neglected in DCM patients. Accordingly, our aims were to (i) evaluate right heart subclinical changes and (ii) the prognostic value of RA compared to left atrial (LA) size and function in patients with DCM by advanced echocardiography. (2) Materials and Methods: Sixty-eight patients with DCM (with a mean age of 60 years; 35 men) were evaluated by comprehensive transthoracic echocardiography, compared to 62 age- and sex-matched healthy controls (with a mean age of 61 years; 32 men), and followed up for 12.4 ± 5 months. (3) Results: DCM patients have RV and RA global longitudinal dysfunction by 2DSTE, higher RA minimum volumes and tricuspid annulus areas despite having normal RV volumes, ejection fractions, and RA maximum volumes by 3DE compared to the controls. The RA strain and RV strain are correlated with each other. The RA reservoir strain (with an AUC = 0.769) has an increased value for outcome prediction compared to that of the LA strain. (4) Conclusion: Patients with DCM have RV longitudinal dysfunction and decreased RA function, in the absence of clinical RV involvement or atrial arrhythmias, and the RA strain is associated with an increased risk of hospitalization and cardiac death.

## 1. Introduction

Dilated cardiomyopathies (DCMs) represent one of the main causes of heart failure (HF) with a reduced ejection fraction (EF) that is mostly diagnosed in the clinical phase of often irreversible myocardial dysfunction represented by reduced left ventricular (LV) EF. The timely detection of subclinical changes during the asymptomatic phase of the disease based on advanced imaging techniques, such as speckle-tracking echocardiography (STE), is of paramount importance for the early implementation of measures that might modify the progression of the disease and decrease the significant morbidity and mortality rates of these patients [1].

While LV dilation and dysfunction have demonstrated prognostic value in DCM and HF with reduced EFs, left atrium (LA) dilation [2], and right ventricular (RV) dilation or dysfunction [3], also significantly impact patients’ prognoses. Furthermore, LV [4], RV, and LA [5] strain analyses are used for the diagnosis of subclinical dysfunction in the risk assessment of patients with DCM, showing an increased value compared to classical functional parameters. However, the right atrial (RA) size and function as well as their clinical implications in DCM are still often neglected, despite the fact that studies have shown their value in predicting adverse outcomes in different cardiac conditions [6,7,8]. Accordingly, our aims were (i) to compare the size and function of the RV, RA, and tricuspid annulus (TA) between patients with DCM without clinical RV involvement or RV pressure/volume overload and healthy subjects by advanced echocardiography techniques; (ii) to evaluate the clinical implications of using conventional versus advanced echocardiography to assess the right heart structure’s size and function in DCM patients for the detection of subclinical cardiac involvement; and (iii) to evaluate the prognostic value of LA and RA size and function in our cohort of patients with DCM.

## 2. Materials and Methods

### 2.1. Study Population

We have retrospectively analyzed prospectively acquired transthoracic echocardiography (TTE) studies of inpatients diagnosed with DCM between July 2021 and October 2022 in the County Clinical Emergency Hospital of Craiova. DCM was defined according to current guidelines as LV dilation and systolic dysfunction in the absence of sufficient abnormal loading conditions or coronary artery disease to cause LV impairment [1]. All patients underwent coronary angiography to exclude the ischemic etiology of LV dilation and dysfunction. The inclusion criteria were as follows: (i) age > 18 years; (ii) diagnosis of DCM; (iii) good quality and complete echocardiographic studies allowing for accurate morpho-functional assessment; and (iv) a minimum follow-up period of 12 months. The exclusion criteria were as follows: (i) clinical RV involvement defined as RV dilation and/or global systolic dysfunction diagnosed according to age- and sex-specific cut-offs of RV volumes and EF derived from three-dimensional echocardiography (3DE) [9]; (ii) sustained supraventricular tachyarrhythmias; (iii) more than mild pulmonary or TR or organic tricuspid valve (TV) disease; (iv) intermediate or high probability of pulmonary arterial hypertension (PAH); and (iv) inadequate acoustic window or poor image quality. The age- and sex-matched healthy subjects included in the control group were chosen from a larger cohort consisting of volunteers as well as adults evaluated as part of a cardiovascular disease screening program. This study was conducted in accordance with the Declaration of Helsinki and approved by the Ethics Committee of The University of Medicine and Pharmacy of Craiova, Romania (No. 241/25.10.2023). Informed consent was obtained from all patients involved in the study upon their admission to the hospital to have their data anonymized and used for research purposes.

### 2.2. Data Collection

The demographical and clinical data for each patient enrolled in the study were collected at the time of inclusion in the study or from the medical records of the patients. All patients underwent complete clinical examination, laboratory tests, and 12-lead ECG recording.

### 2.3. Echocardiographic Acquisitions and Analysis

**Figure 1:**
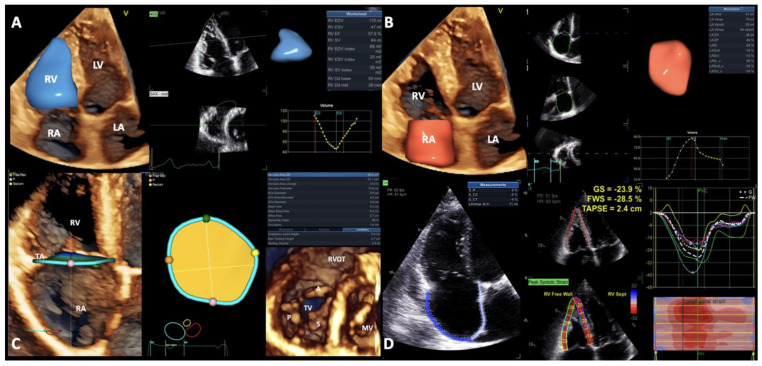
Right heart chamber and TA evaluation by 3DE and 2DSTE using dedicated software packages (EchoPac v204, GE, Horten, Norway). (**A**) RV volumes and function assessment using 4D Auto RVQ; (**B**) RA volume and function assessment by 4D Auto LAQ; (**C**) Tricuspid valve complex evaluation using 4D Auto TVQ; and (**D**) RA and RV strain analysis by AFI LA and AFI RV. Abbreviations: 2Ch—two chamber, 2D—two-dimensional, 2DSTE—two-dimensional speckle-tracking echocardiography, 3DE—three-dimensional speckle-tracking echocardiography, 4Ch—4 chamber, A—anterior leaflet, ED—end-diastole, ES—end-systole, FWS—free-wall strain, GS—global strain, LA—left atrium, LV—left ventricle, MV—mitral valve, P—posterior leaflet, PreA—before atrial contraction, PVC—pulmonary valve closure, RA—right atrium, RV—right ventricle, RVEDV—right ventricular end-diastolic volume, RV EF—right ventricular ejection fraction, RVESV—right ventricular end-systolic volume, RVOT—right ventricular outflow tract, S—septal leaflet, S_CD—conduit strain, S_CT—contractile strain, S_R—reservoir strain, TA—tricuspid annulus, and TV—tricuspid valve.

All patients underwent clinically indicated comprehensive two-dimensional (2DE), Doppler echocardiography, two-dimensional STE (2DSTE), and 3DE using commercially-available Vivid E95 (GE Vingmed, Horten, Norway) scanner equipped with 4Vc probe. All patients were hemodynamically stable and compensated after diuretic treatment optimization at the time of the TTE examination. The standard echocardiographic measurements were performed according to current guidelines, and the advanced echocardiographic measurements were obtained using the software packages included in EchoPAC v204, GE, Horten, Norway by the offline analysis of digitally stored datasets (Figure 1).

To confirm the presence of LV dilation and dysfunction, LV size and EF were measured by the 2DE biplane method of disk summation using the apical 2-chamber and 4-chamber views and by the 3DE software package 4D AutoLVQ (EchoPAC v204) using the 3D multibeat datasets of the LV [9]. LV global longitudinal strain was measured from the apical 2-, 3-, and 4-chamber non-foreshortened views using the dedicated software package AFI LV (EchoPAC v204).

The probability of PAH was checked using the maximum velocity of the continuous-wave doppler signal of the TR jet and the presence or absence of other echocardiographic signs of PAH [10]. The presence and the severity of valvular regurgitations were evaluated using the multi-parametric algorithm recommended in the latest European guidelines [11], and the absence of structural TV disease was confirmed by obtaining multiple cut-planes from the volume-rendered 3DE dataset of the valve. The size and shape of the TV complex was evaluated using a dedicated software package (4D AutoTVQ, EchoPAC v204) and included the 3DE and 2DE tricuspid annulus (TA) areas, TA area change, TA diameters, and TV tenting height and volume.

The assessment of RV size and function by conventional echocardiography included RV areas measured at end-diastole (ED) and end-systole (ES) in the apical RV-focused view and the subsequently derived RV fractional area change (FAC), as well as the TA plane systolic excursion (TAPSE) by M-mode echocardiography in the apical 4-chamber view [9]. The advanced evaluation of RV dimensions and function included the measurement of the RV free-wall longitudinal strain (RVFWLS) by 2DSTE in the RV focused apical view using a dedicated software package (AFI RV, EchoPAC v204) as well as RV volumes at ED (EDV) and ES (ESV), RV stroke volume (SV), and RV EF by 3DE using a dedicated software package (4D AutoRVQ, EchoPAC v204).

The RA maximum volume (Vmax) was measured by 2DE using the area length method in the RV-focused apical view [9,12] and also by 3DE together with the RA minimum volume (Vmin), emptying fraction (EmF), and emptying volume (EV) by adapting a software package dedicated for the LA (4D AutoLAQ, EchoPAC v204) to the RA. The same software was used for the LA evaluation by 3DE. Finally, the LA and RA global longitudinal strain in the reservoir, conduit, and contractile phases was calculated from the apical 2- and 4-chamber views for the LA and from the RV focused apical view for the RA using different software for the LA (AFI LA, EchoPAC v204). The automatically-derived LA Vmax by the 2DSTE AFI LA software was used instead of the 2DE LA volumes [13]. Absolute values were reported for all strain parameters to facilitate their statistical comparison. The dimensional parameters were indexed to the body surface area (BSA).

### 2.4. Follow Up and Endpoints of the Study

A combined endpoint of cardiac death and rehospitalization for HF decompensation was chosen. Information regarding patient survival and rehospitalization was obtained via telephone with the patient or their family members and review of the electronic hospital admission records. Mortality status was verified independently, and cardiac cause of death was confirmed based on post mortem exam reports when available or medical records of the patients who died while hospitalized. For patients without events, the date of the last contact was used for survival analysis.

### 2.5. Statistical Analysis

The normal distribution of the variables was confirmed using the Kolmogorov–Smirnov test. Continuous variables are reported as mean ± standard deviation (SD), and categorical ones are reported as number and percentage. The student’s *t*-test for independent variables was used for inter-group comparison. Pearson’s correlation coefficient was used to assess the correlations between variables. Cox proportional hazard model was used to determine the association between echocardiographic data (including basic and advanced parameters of left and right atrial and ventricular function) and the outcome. Receiver operator characteristic (ROC) curves were used to compare the predictive prognostic value of the echocardiographic parameters that were associated with the combined endpoint for the Cox analysis. The statistical significance of the differences between the areas under the curve (AUC) resulting from the corresponding ROC curves was assessed by ROC analysis. A *p* value < 0.05 was considered statistically significant. The statistical analysis was performed using SPSS version 23 for Mac (SPSS Inc., IBM Corp., Chicago, IL, USA). The reproducibility of the echocardiographic measurements has been reported elsewhere.

## 3. Results

From the 106 patients with DCM analyzed, six patients were excluded due to clinical RV involvement, eight patients due to having more than mild TR, four due to an intermediate or high likelihood of PAH, eight due to the presence of atrial fibrillation (AF) or atrial flutter, seven due to incomplete or inadequate 3DE datasets, and five due to a lack of follow-up data. The final study population consisted of 68 (with a mean age of 60 years; 35 men) patients with DCM who were compared to 62 age- and sex-matched healthy subjects (with a mean age of 61 years; 32 men). The demographic, clinical, and paraclinical data of the patients with DCM in comparison with the healthy volunteers are summarized in Table 1.

### 3.1. Assessment of RV Size and Function (Table 2)

Although patients with DCM had normal RV volumes and EF by 3DE, the RV EDV (44 ± 15 versus 53 ± 11 mL/m^2^, *p* = 0.002, Table 2) and SV (23 ± 9 versus 31 ± 6 mL/m^2^, *p* < 0.001) indexed to BSA, as well as the RV EF (53 ± 9 versus 59 ± 6%, *p* < 0.001) were significantly lower in the DCM group compared to the controls.

RV size and function assessed by the 2DE-derived RV areas at both ED and ES and RV FAC were similar between the patients with DCM and the subjects in the control group (*p* > 0.05 for all).

Furthermore, while patients with DCM had normal longitudinal function evaluated as TAPSE by M-mode, the values of TAPSE were significantly lower in DCM patients compared to those of the control group (19 ± 2 versus 23 ± 2, *p* < 0.001). Conversely, when assessed by 2DSTE, the DCM patients had longitudinal dysfunction and significantly reduced RVFWLS values (19 ± 6 versus 32 ± 3%, *p* < 0.001) compared to the controls.

**Table 2:** Right heart structure’s size and function evaluation by conventional and advanced echocardiography.

Parameter	DCM Patients (*n* = 68)	Healthy Subjects (*n* = 62)	*p* Value
TAPSE (mm)	19 ± 2	23 ± 2	<0.001
RV EDA indexed to BSA (cm^2^/m^2^)	12 ± 4	11 ± 2	0.224
RV ESA indexed to BSA (cm^2^/m^2^)	6.4 ± 3	5.6 ± 1	0.055
RV FAC (%)	46 ± 8	48 ± 5	0.056
RV FWLS (%)	19 ± 6	32 ± 3	0.012
RV EDV indexed to BSA (mL/m^2^)	44 ± 15	53 ± 11	0.002
RV ESV indexed to BSA (mL/m^2^)	21 ± 8	22 ± 6	0.413
RV SV indexed to BSA (mL/m^2^)	23 ± 9	31 ± 6	<0.001
RV EF (%)	53 ± 9	59 ± 6	<0.001
2D RA Vmax indexed to BSA (mL/m^2^)	25 ± 11	29 ± 6	0.042
3D RA Vmax indexed to BSA (mL/m^2^)	29 ± 12	29 ± 6	0.785
3D RA Vmin indexed to BSA (mL/m^2^)	16 ± 8	12 ± 5	0.015
3D RA EmF (%)	44 ± 20	58 ± 9	<0.001
3D RA EmV (mL)	25 ± 19	30 ± 7	0.114
RA global longitudinal strain in the reservoir phase (%)	26 ± 11	40 ± 10	<0.001
RA global longitudinal strain in the conduit phase (%)	11 ± 7	15 ± 10	<0.001
RA global longitudinal strain in the contractile phase (%)	16 ± 8	25 ± 9	<0.001
RA Vmin/RV EDV	0.39 ± 0.2	0.2 ± 0.1	<0.001
3D TA area indexed to BSA (cm^2^/m^2^)	5.5 ± 1.2	4.7 ± 1.1	0.002
2D TA area indexed to BSA (cm^2^/m^2^)	4.9 ± 1.2	4.6 ± 1.1	0.234
TA area change (%)	16 ± 6.5	19 ± 6.7	0.063
TA perimeter indexed to BSA (cm/m^2^)	5.8 ± 1	6.1 ± 1	0.234
TV 4Ch diameter indexed to BSA (cm/m^2^)	1.9 ± 0.3	1.9 ± 0.4	0.719
TV major diameter indexed to BSA (cm/m^2^)	2.6 ± 3.1	2 ± 0.4	0.273
TV minor diameter indexed to BSA (cm/m^2^)	1.6 ± 0.3	1.7 ± 0.3	0.061
TV tenting height (cm)	0.7 ± 0.2	0.6 ± 0.1	0.197
TV tenting volume (mL)	1.9 ± 1.2	1.4 ± 0.7	0.088

Abbreviations: 4Ch, apical 4-chamber view; BSA, body surface area; DCM, dilated cardiomyopathy; EDA, end-diastolic area; EmF, emptying fraction; ESA, end-systolic area; FAC, fractional area change; FWLS, free-wall longitudinal strain; RV, right ventricle; SV, stroke volume; TA, tricuspid annulus; and TV, tricuspid valve. Other abbreviations as in Table 1.

### 3.2. Assessment of RA Size and Function (Table 2)

The RA Vmax indexed to BSA measured by 2DE at ES was higher in the control group compared to the patients with DCM (29 ± 6 versus 25 ± 11 mL/m^2^, *p* = 0.042).

By 3DE, the RA Vmax was shown to be similar between the groups (29 ± 12 versus 29 ± 6 mL/m^2^, *p* = 0.785), yet the RA Vmin was significantly larger (16 ± 8 versus 12 ± 5 mL/m^2^, *p* = 0.015) and the RA EmF (44 ± 20 versus 58 ± 9%, *p* < 0.001) was significantly lower in DCM patients compared to the control group.

The RA function evaluated by 2DSTE as the RA strain in the reservoir (26 ± 11 versus 40 ± 10%, *p* < 0.001), conduit (11 ± 7 versus 15 ± 10%, *p* < 0.001), and contractile (16 ± 8 versus 25 ± 9%, *p* < 0.001) phases was significantly reduced in the DCM group compared to the healthy controls during all three phases.

Finally, the RA-to-RV coupling index measured as RAVmin/RVEDV had significantly higher values in the DCM group (0.39 ± 0.2 versus 0.2 ± 0.1, *p* < 0.001), suggesting initial RA-to-RV uncoupling in these patients.

### 3.3. Assessment of TA Size and Function (Table 2)

There were no statistically significant differences in terms of TV and TA linear measurements or the 2DE TA area between the two groups, while the 3D TA area (5.5 ± 1.2 versus 4.7 ± 1.1 cm^2^/m^2^, *p* = 0.002) was significantly larger in the DCM group.

### 3.4. Correlations between the Echocardiographic Parameters

The 3D TA area was correlated with the majority of RA and RV volumetric and functional parameters by both 2DSTE and 3DE, as well as with the LV volumes and EF by 3DE. Furthermore, as expected, the RA and RV parameters were correlated with each other (Table 3).

### 3.5. Prognostic Value of LA and RA Size and Function

During a mean follow up of 12.4 ± 5 months, 27 (39.7%) of the patients reached the composite outcome of HF rehospitalization or cardiac death. The clinical and echocardiographic characteristics of the study population dichotomized based on the occurrence of events are represented in Table 4. There were no statistically significant differences in terms of the occurrence of events based on the clinical characteristics of the patients, including guideline-directed medical therapy for HF with a reduced EF. Regarding the echocardiographic data, we found that patients with events had more moderate to severe levels of MR (*p* = 0.004) and decreased RA function (*p* = 0.006 for RA reservoir strain, *p* = 0.026 for RA contractile strain, and *p* = 0.011 for RA EmF).

LA and RA size and function parameters and their relation with the outcome were tested in a Cox regression analysis (Table 5). The parameters that were strongly related to the outcome were the LA reservoir strain (Beta = 1.053, *p* = 0.049), RA reservoir strain (Beta = 1.054, *p* = 0.012), and RA EmF (Beta = 1.022, *p* = 0.041).

**Figure 2:**
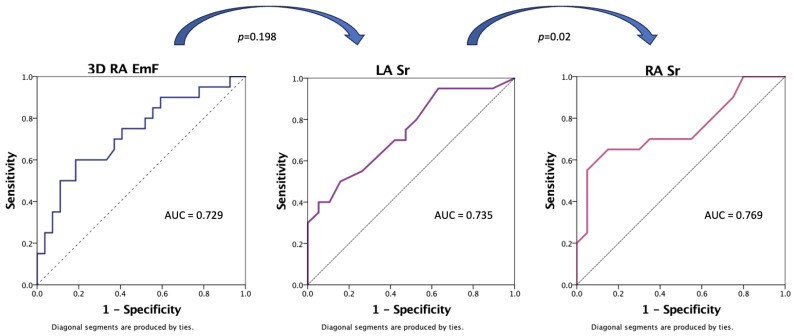
Receiving operator characteristic curves and analysis for comparing the prognostic value of RA and LA function parameters associated with the outcome. Abbreviations: AUC, area under the curve; EmF, emptying fraction; LA, left atrium; RA, right atrium; and Sr, reservoir strain.

When evaluated in ROC curves (Figure 2), the greatest predictive value for the composite of cardiac death and HF rehospitalization by ROC analysis was found for the RA reservoir strain (AUC = 0.769, *p* = 0.02 compared to the LA reservoir strain with an AUC = 0.735).

## 4. Discussion

To the best of our knowledge, our study is the first to use state-of-the-art advanced echocardiography techniques to compare right heart chamber and TA remodeling and function in patients with DCM without clinical RV involvement or RV pressure or volume overload with age- and sex-matched healthy controls. Our main results can be summarized as follows: (a) DCM patients without clinical RV involvement, PAH, significant TR, or sustained atrial arrhythmias have (i) lower RV EDV and global systolic function when assessed by 3DE, (ii) RV longitudinal dysfunction by 2DSTE, and (iii) similar RA Vmax, larger RA Vmin, and TA area values by 3DE and reduced RA function by 3DE and 2DSTE compared to age- and sex-matched controls; (b) (i) RA strain and RV strain are correlated with each other and (ii) TA size is correlated with RVFWLS and RA contractile strain by 2DSTE, RA Vmax, and EV, RV, and LV volumes and LVEF by 3DE; (c) when using classical echocardiographic parameters for the evaluation of right heart structure remodeling and function in patients with DCM compared to healthy subjects, these differences are overlooked; and (d) the RA reservoir strain was independently associated with the outcome and had a greater prognostic value compared to the LA reservoir strain and RA EmF in our cohort of patients.

The patients with DCM included in our study had significantly different right heart chambers and TA sizes and function when assessed by advanced TTE techniques compared to the control group; however, the only parameters that fell outside the reference ranges were the RVFWLS, RA Vmin, RA EmF, RA reservoir, conduit and contractile strain, and 3D TA area.

RV assessment in patients with DCM by either echocardiography or more advanced imaging techniques is mandatory at diagnosis and during follow up due to the prognostic implications of both subclinical and clinical RV dilation and dysfunction in these patients. Several components contribute to the global systolic function of the RV: the contraction of the longitudinal fibers which leads to RV shortening and the motion of the TA towards the apex, the inward motion of the RV free-wall that occurs as a results of the contraction of the circumferential ones, the infundibular contraction, and finally the contraction of the LV via the interventricular septum as part of ventricular interdependence [3]. In patients with DCM, their RV dysfunction results from either direct RV myocardial involvement, pressure, or volume overload, or a combination of these, mostly depending on the underlying etiology [14,15], and has a reported prevalence of 34–65% [16,17]. In our study, we have chosen DCM patients without RV dilation and dysfunction assessed by 3DE, which provides excellent accuracy compared the gold-standard cardiac magnetic resonance (CMR) [18], significant tricuspid regurgitation (defined as more than mild), and an intermediate or high probability of PAH as evident causes of RV volume or pressure overload, as well as DCM patients without a documented current or past history of sustained atrial tachyarrhythmias because of their impact on RV function [19] by reducing the RV preload. We found that RV longitudinal myocardial deformation is impaired in DCM patients, who have a reduction in RV EF compared to the control group. However, when assessing it by M-mode or 2DE, RV function was normal in both groups despite the fact that TAPSE had lower values in the DCM patients compared to the control group. These findings can be explained by the well-known limitations of conventional RV functional parameters—TAPSE evaluates the longitudinal function only at the level of the basal RV free-wall and is angle- and load-dependent, and RV FAC, although it evaluates RV global systolic function, neglects the contribution of the RV inflow tract to the global contraction [9]. Accordingly, we have demonstrated that the use of advanced echocardiography is of paramount importance for the detection of early RV involvement in DCM patients, especially because of the demonstrated prognostic value of RVFWLS evaluation in several studies [20,21].

On the other hand, the RA remains the most neglected cardiac chamber, and its dimensional and functional evaluation is overlooked in the vast majority of cardiac conditions. Only recently have assessments of RA and consequently of TA remodeling gained interest, particularly in patients with AF or PAH [7,22]. RA structural, electrical, and metabolic remodeling have been described in patients with AF, and RA volume overload in patients with significant secondary TR plays a key role in the pathophysiological cascade leading to these RA changes [23]. Accordingly, we have excluded patients with DCM and a current or documented history of supraventricular arrhythmias, as well as more than mild TR, to evaluate the changes in RA size and function in patients with isolated left-sided clinical disease. We have demonstrated that the RA function assessed by either 2DSTE as RA strain or 3DE as RA EmF is reduced in DCM compared to that of the control group despite normal RA Vmax values. RA structure and function by CMR are impaired in patients with DCM, and the RA reservoir and conduit strain were demonstrated to be independently associated with adverse clinical outcomes and to have a higher predictive value compared to RA EmF [24]. However, these findings were demonstrated on Chinese patients, and geographical variations have been found in both RA size and function [25]. In our study, we found that RA Vmax is lower in DCM patients compared to the control group when measured by 2DE and similar between groups when measured by 3DE. Despite the fact that we measured the RA Vmax in the RV-focused apical view in order to obtain a more accurate RA quantification by 2DE and similar RA Vmax values by 2DE and 3DE [12], our results show that in DCM patients, the RA size assessment by 2DE might not provide the same results as 3DE. We hypothesize that these differences might be explained by the fact that in patients with DCM, the RV-focused apical view cannot be obtained as correctly as in healthy subjects, therefore leading to the underestimation of the size of the right heart chambers by 2DE compared to 3DE. Finally, the RA Vmin values together with the TA area were significantly larger in DCM patients when evaluated by 3DE compared to those of the healthy subjects. The changes in TA area are probably secondary to the RA Vmin increase in the DCM group, as RA Vmin has been demonstrated to be the main determinant of TA area [22], although in our study, the TA area was correlated with RA, RV, and LV size and function by 2DSTE or 3DE.

RA remodeling is associated with a negative prognosis and disease severity in many cardiac conditions. In HF patients undergoing cardiac resynchronization therapy [26,27], RA volume predicted patient mortality, and it was correlated with increased RA pressures. Furthermore, RA strain by CMR is an independent predictor of the outcome in patients with systemic sclerosis [28] and precapillary pulmonary hypertension [29]. However, the predictive value of RA strain was less tested in isolated LV dilated cardiomyopathy patients. Accordingly, we have shown that RA reservoir strain is a valuable parameter for the prognostic evaluation of patients with DCM and without clinical RV involvement, with a superior value compared to that of LA strain for event prediction.

Our study has important clinical implications because we have shown that patients with DCM have RA and RV involvement without a secondary cause that might explain the subclinical RA or RV dysfunction. While RV assessment in patients with DCM is mostly performed, RA size and especially RA function are very rarely reported and might provide significant prognostic information, as shown by our paper. However, existing data on the intrinsic RA cardiomyopathic process are scarce, and our results should be further demonstrated in larger cohorts and different clinical scenarios.

### Limitations

We acknowledge several limitations of our study. First, we have included a relatively reduced number of patients from a single center mainly due to the need to have an accurate phenotypical characterization of the study population and to avoid the confounding effect of coexisting pathological conditions. In addition, due to the retrospective nature of the study, there might have been selection bias. Second, we have used specific software packages for both the 2DSTE and 3DE evaluations of the RV, RA, and TA, and our results might not be reproduced when using other available software packages. Third, we did not evaluate the changes in the size and function of the right heart chambers based on the etiology of DCM or RV diastolic function. Fourth, we cannot exclude undetected episodes of AF that might have led to RA dysfunction in our cohort of patients or the impact of associated cardiovascular risk factors.

## 5. Conclusions

Patients with DCM are shown have RV longitudinal dysfunction and decreased RA function by 2DSTE and 3DE, and similar RA Vmax and larger RA Vmin and TA areas by 3DE compared to those of age- and sex-matched healthy subjects, in the absence of RV dilation or global systolic dysfunction, RV pressure or volume overload, or sustained atrial arrhythmias. RA strain and RV strain are correlated with each other, and the 3D TA area correlates with the RA contractile strain and RVFWLS, RA Vmax, RV and LV volumes, and LV EF in patients with DCM. The RA reservoir strain was associated with the outcome and a had greater predictive value compared to the LA strain in our cohort of patients. An RA strain reduction might suggest an initial RA cardiomyopathic process in patients with DCM, and the early detection of RA dysfunction might help reduce patients’ risk and stratify the patients in need of closer follow ups and monitoring.

## Figures and Tables

**Table 1:** Comparison between the demographic, clinical, and left heart echocardiographic data of the DCM group and healthy subjects.

Parameter	DCM Patients (*n* = 68)	Healthy Subjects (*n* = 62)	*p* Value
Age (years)	60 ± 10	61 ± 8	0.162
BSA (m^2^)	1.9 ± 0.2	1.94 ± 1.6	0.007
Heart rate (beats per minute)	74 ± 14	77 ± 16	0.030
Systolic blood pressure (mmHg)	115 ± 10	120 ± 15	0.065
Diastolic blood pressure (mmHg)	69 ± 8	70 ± 6	0.501
Arterial hypertension	21 (30.9%)	0 (0%)	<0.001
Smoking	11 (16.2%)	0 (0%)	<0.001
Type 2 diabetes mellitus	16 (23.5%)	0 (0%)	<0.001
Chronic kidney disease	9 (13.2%)	0 (0%)	<0.001
Of which with Type 2 diabetes mellitus	5 (7.4%)	0 (0%)	<0.001
Of which with an eGFR < 30 mL/min/1.73 m^2^	8 (11.8%)	0 (0%)	<0.001
Dyslipidemia	34 (50%)	0 (0%)	<0.001
Implantable cardiac defibrillator	4 (5.9%)	0 (0%)	<0.001
Tricuspid regurgitation grading			
Absent or trivial	26 (38.2%)	50 (80.6%)	<0.001
Mild	21 (30.9%)	12 (19.4%)	<0.001
Mitral regurgitation grading			
Absent	10 (14.7%)	62 (100%)	<0.001
Mild	18 (24.5%)	0 (0%)	<0.001
Moderate	9 (13.2%)	0 (0%)	<0.001
Severe	10 (16.6%)	0 (0%)	<0.001
3D LV EDV indexed to BSA (mL/m^2^)	103 ± 4	52 ± 5	<0.001
3D LV ESV indexed to BSA (mL/m^2^)	73 ± 3	19 ± 3	<0.001
3D LV EF (%)	33 ± 2	59 ± 5	<0.001
LV global longitudinal strain (%)	8.6 ± 2.8	22 ± 2	<0.001
3D LA Vmax indexed to BSA (mL/m^2^)	40 ± 11	27 ± 7	<0.001
2DSTE LA Vmax indexed to BSA (mL/m^2^)	37 ± 14	26 ± 8	<0.001
2D LA Vmax indexed to BSA (mL/m^2^)	28 ± 11	24 ± 6	<0.001
LA global longitudinal strain in the reservoir phase (%)	16 ± 8	41 ± 8	<0.001
LA global longitudinal strain in the conduit phase (%)	8 ± 4	26 ± 10	<0.001
LA global longitudinal strain In the contractile phase (%)	8 ± 6	15 ± 5	<0.001

Abbreviations: 2D, two-dimensional; 2DSTE, two-dimensional speckle-tracking echocardiography; 3D, three-dimensional; BSA, body surface area; DCM, dilated cardiomyopathy; EDV, end-diastolic volume; EF, ejection fraction; ESV, end-systolic volume; GFR, glomerular filtration rate; LA, left atrium; LV, left ventricle; Vmax, maximum volume.

**Table 3:** Correlations between the echocardiographic parameters.

3D TA area									
	3D RA Vmax	RA EV	3D TA area change	RV EDV		RV ESV	3D LV EF	3D LV EDV	3D LV ESV
r	0.513	0.410	−0.544	0.404		0.444	−0.361	0.394	0.355
*p*	<0.001	0.003	0.001	0.004		0.001	0.028	0.016	0.031
RV FWLS									
	RA S_r	RA S_cd	RA Vmax	3D TA area		RV EDV	RV ESV		
r	0.360	0.347	−0.306	−0.373		−0.419	−0.407		
*p*	0.013	0.017	0.031	0.008		0.002	0.003		
RA S_r									
	RA S_cd	RA S_ct	RA Vmax	RA Vmin					
r	0.630	0.857	−0.433	−0.311					
*p*	<0.001	<0.001	0.002	0.034					
RA S_ct						3D RA Vmin			
	TA area	RV EDV	RV ESV			RV EF	3D RA Vmax	RA EmF	3D TA area change
r	−0.293	−0.341	−0.349		r	−0.322	0.601	−0.630	−0.429
*p*	0.046	0.019	0.016		*p*	0.012	<0.001	<0.001	0.013

Abbreviations: S_cd, conduit strain; S_ct, contractile strain; and S_r, reservoir strain. Other abbreviations as in Table 1 and Table 2.

**Table 4:** Clinical and echocardiographic characteristics based on the outcome.

Parameter	DCM Patients with Events (*n* = 27)	DCM Patients without Events (*n* = 41)	*p* Value
Demographic and clinical data			
Age (years)	62 ± 9	58 ± 11	0.171
BSA (m^2^)	1.24 ± 0.43	1.27 ± 0.45	0.371
Heart rate (beats per minute)	74 ± 13	75 ± 15	0.763
Systolic blood pressure (mmHg)	117 ± 12	114 ± 7	0.412
Diastolic blood pressure (mmHg)	70 ± 8	69 ± 8	0.761
Arterial hypertension	(47.6%)	(42.3%)	0.723
NYHA class ≥ III	(58%)	(51%)	0.057
Smoking	(19%)	(26.9%)	0.536
Type 2 diabetes mellitus	(23.8%)	(42.3%)	0.191
Chronic kidney disease	(19%)	(19.2%)	0.988
Dyslipidemia	(61.9%)	(80.8%)	0.168
Implantable cardiac defibrillator	(14.3%)	(3.8%)	0.251
ACE-I/ARNI	(73%)	(90.5%)	0.150
Betablockers	(92.3%)	(100%)	0.161
Mineralocorticoid receptor antagonists	(80.8%)	(90.5%)	0.363
SGLT2i	(38.5%)	(42.9%)	0.766
Guideline-directed medical therapy (all four classes)	(30.8%)	(38.1%)	0.608
Diuretics	(65.4%)	(85.7%)	0.117
Echocardiographic data			
Tricuspid regurgitation grading			
Absent or trivial	(52.4%)	(57.7%)	0.828
Mild	(47.6%)	(42.3%)	0.826
Mitral regurgitation grading			
Absent or mild	(47.6%)	(69.3%)	0.042
Moderate to severe	(52.4%)	(30.7%)	0.004
3D LV EDV indexed to BSA (mL/m^2^)	108 + 21	100 ± 19	0.263
3D LV ESV indexed to BSA (mL/m^2^)	76 ± 19	70 ± 16	0.348
3D LV EF (%)	32 ± 7	35 ± 14	0.429
TAPSE (mm)	17 ± 4	18 ± 4	0.826
RV EDA indexed to BSA (cm^2^/m^2^)	12 ± 5	11 ± 4	0.599
RV ESA indexed to BSA (cm^2^/m^2^)	7 ± 3	6 ± 2	0.686
RV FAC (%)	46 ± 8	45 ± 8	0.856
RV FWLS (%)	19 ± 6	20 ± 6	0.649
RV EDV indexed to BSA (mL/m^2^)	44 ± 18	44 ± 14	0.970
RV ESV indexed to BSA (mL/m^2^)	20 ± 9	21 ± 9	0.815
RV SV indexed to BSA (mL/m^2^)	24 ± 11	23 ± 8	0.891
RV EF (%)	53 ± 9	53 ± 10	0.954
2D RA Vmax indexed to BSA (mL/m^2^)	24 ± 11	26 ± 11	0.405
3D RA Vmax indexed to BSA (mL/m^2^)	29 ± 11	29 ± 12	0.970
3D RA Vmin indexed to BSA (mL/m^2^)	18 ± 8	13 ± 8	0.070
3D RA EmF (%)	37 ± 17	52 ± 21	0.011
3D RA EmV (mL)	21 ± 16	30 ± 22	0.088
RA global longitudinal strain in the reservoir phase (%)	17 ± 9	27 ± 12	0.006
RA global longitudinal strain in the conduit phase (%)	10 ± 9	14 ± 10	0.227
RA global longitudinal strain in the contractile phase (%)	8 ± 7	14 ± 9	0.026
RA Vmin/RV EDV	0.43 ± 0.2	0.35 ± 0.2	0.222
3D TA area indexed to BSA (cm^2^/m^2^)	5.8 ± 1.3	5.3 ± 1.1	0.127
2D TA area indexed to BSA (cm^2^/m^2^)	5.1 ± 1.3	4.6 ± 1.1	0.355

Abbreviations: ACE-I, angiotensin converting enzyme inhibitors; ARNI, angiotensin receptor/neprilysin inhibitor; NYHA, New York Heart Association; and SGLT2i, sodium–glucose co-transporter-2 inhibitors. Other abbreviations as in Table 1 and Table 2.

**Table 5:** Univariate Cox regression analysis testing the correlation between the echocardiographic variables and the combined endpoint in patients with DCM.

Parameter	Beta	*p* Value
TAPSE (mm)	0.981	0.752
RV FAC (%)	0.983	0.587
RV FWLS (%)	0.965	0.385
3D RA Vmax indexed to BSA (mL/m^2^)	0.993	0.724
RA global longitudinal strain in the reservoir phase (%)	1.052	0.012 *
RA global longitudinal strain in the conduit phase (%)	1.022	0.299
RA global longitudinal strain in the contractile phase (%)	1.028	0.051
RV EDV indexed to BSA (mL/m^2^)	0.993	0.653
RV ESV indexed to BSA (mL/m^2^)	0.985	0.589
RV EF (%)	1.001	0.968
3D RA Vmax indexed to BSA (mL/m^2^)	0.996	0.859
3D RA Vmin indexed to BSA (mL/m^2^)	0.949	0.110
3D RA EmF (%)	1.022	0.041 *
3D LV EF (%)	1.036	0.193
3D LV EDV indexed to BSA (mL/m^2^)	1.015	0.372
3D LV ESV indexed to BSA (mL/m^2^)	1.004	0.822
LA global longitudinal strain in the reservoir phase (%)	1.053	0.049 *
LA global longitudinal strain in the conduit phase (%)	1.019	0.752
LA global longitudinal strain in the contractile phase (%)	1.041	0.382
3D LA Vmax indexed to BSA (mL/m^2^)	1.000	0.991
3D LA Vmin indexed to BSA (mL/m^2^)	0.989	0.653
3D LA EmF (%)	1.013	0.481

Abbreviations: as in the other tables; * statistically significant.

## Data Availability

The data presented in this study are available upon request from the corresponding author, following the approval from the University of Medicine and Pharmacy of Craiova, Romania.
